# Combustion Synthesis Porous Nitinol for Biomedical Applications

**DOI:** 10.1155/2019/4307461

**Published:** 2019-04-03

**Authors:** H. Aihara, J. Zider, G. Fanton, T. Duerig

**Affiliations:** ^1^PorOsteon Spine Inc., Menlo Park, California, USA; ^2^Department of Orthopedic Surgery, Sports Medicine Division, Stanford University Medical Center, Palo, Alto, California, USA; ^3^Confluent Medical Technologies, Fremont, California, USA

## Abstract

Porous Nitinol with a three-dimensional anisotropic interconnective open pore structure has been successfully produced by the combustion synthesis (CS) of elemental Ni and Ti powders. The resulting product can be tailored to closely match the stiffness of cancellous bone to minimize stress shielding. The average elastic modulus was approximately 1 GPa for a porosity of 60 vol% and the average pore size of 100-500 *µ*m. The low elastic modulus meets the basic demand for orthopedic bone ingrowth applications. Furthermore, porous Nitinol was composed of cubic (austenitic) and monoclinic (martensitic) NiTi compounds without the presence of Ni metal or Ni-rich phases. The resulting product exhibits excellent corrosion resistance with breakdown potentials above 750mV. An ovine study in cortical sites of the tibia demonstrated rapid osseointegration into the porous strucutre as early as two weeks and complete bone growth across the implant at six weeks. A separate ovine study showed complete through-growth of bone at four months using a lumbar interbody fusion model, substantiating the use of porous Nitinol as an implant material for applications in the spine. Porous Nitinol is thus a promising biomaterial with proven biocompatibility and exceptional osseointegration performance which may enhance the healing process and promote long-term fixation, making it a strong candidate for a wide range of orthopedic implant applications.

## 1. Introduction

Since the early 1960s, orthopedic implants were made from three distinct materials types, metals, ceramics, and polymers. Commonly used materials are cobalt base metal alloys, stainless steel, titanium, polyetheretherketon (PEEK), ziroconia and alumina ceramics, poly l'lactic acid (PLLA), or poly glycolic acid (PGA) biodegradable materials. In spine, solid titanium has historically been used for spinal fusion implants. Since the early 2000s, PEEK was introduced and gained acceptance for its low modulus and radiolucent properties [1, 2]; however, recent studies have shown that PEEK does not integrate well with surrounding bone and may form a fibrous connective interface. Due to its hydrophobic and bioinert properties, the spine industry is undergoing a paradigm shift, moving back to metals to improve osseointegration at the bone-to-implant interface and to increase long-term implant stability [3].

Nitinol has widely been used for decades in cardiovascular, neurological and orthopedic devices. In its wrought state, Nitinol exhibits a modulus of elasticity of between 40-75 GPa depending upon its transformation temperature and crystallographic texture. In contrast, wrought stainless steel and titanium exhibit moduli of up to 210 and 110 GPa, respectively [4]. While the elastic modulus of cortical bone is between 17-20 GPa, that of cancellous is approximately 1.15 GPa [5–7]. The implantation of solid titanium or stainless-steel devices can lead to stress shielding due to the mismatch of stiffness between the implant and the surrounding bone. “Stress shielding” refers to the slow healing and reduction of bone density that results when implants remove the stresses normally experienced by bone. Stress shielding is minimized by using implants that exhibit stiffness similar to the surrounding bone. Stiffness, in turn, is influenced by both the design and by the elastic modulus of the implant material. The development of biocompatible materials that closely match the mechanical properties of bone is thus important to improving patient outcomes in a variety of orthopedic procedures. But the inherent difference in moduli between bone and any wrought metal is vast, and cannot be bridged by conventional means.

One approach that has been successfully used to increase implant compliance is to synthesize highly porous implants. In addition to lowering the effective modulus, porous materials can dramatically improve boney ingrowth and surface friction. The early introduction of porous metals was in the form of coatings, as beads and wire mesh that had been sintered or metallic particles plasma sprayed on the solid implant surface [8]. Fully porous metals have been employed in orthopedic applications within the past several years with introduction of porous tantalum, known as Trabecular Metal™ (Zimmer), and porous titanium such as Regenerex® (Biomet), Biofoam™ (Wright Medical), and Tritanium™ (Stryker) [9].

The cost of implants is always a top concern for new technologies. Both porous tantalum and porous titanium require a significant capital investment. Trabecular Metal™ is manufactured by physical vapor deposition (PVD) which requires long processing time, while the cost of 3D printed implants made from porous titanium by additive manufacturing are influenced by its printing speed, the size of print space, and the high cost of powder specific to the machine [10]. Both processes share the speed of manufacturing as the limiting factor which inflate the manufacturing cost; adding to the cost of implants. Porous Nitinol on the other hand exhibits cost advantage for its speed of manufacturing and the need for low capital investment.

**Figure 1:**
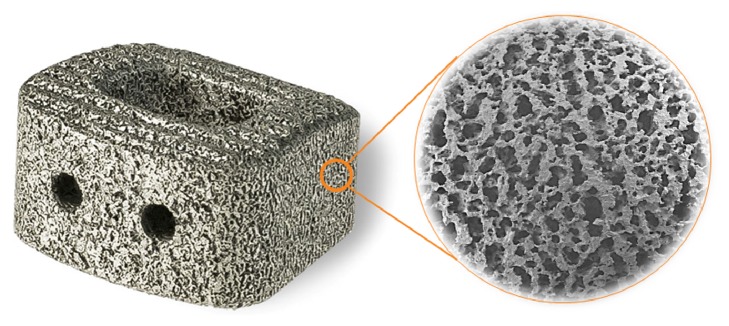
Commercially available porous Nitinol cage for anterior cervical intervertebral fusion and SEM image displaying the irregular pore morphology.

Nitinol is an intermetallic compound and thus a candidate for a unique and new route to forming a porous biomaterial: combustion synthesis (CS). The CS process takes advantage of the exothermic chemical reaction between two or more elemental powders to produce an intermetallic compound—in this case, nickel and titanium reacting to form the equiatomic TiNi compound [11]. Once the reaction is initiated, a high temperature reaction flame front propagates through the material, synthesizing the TiNi compound from the elemental powders in a matter of seconds. The heat of formation of Ti+Ni->TiNi reaction is 67.8KJ/mole [12]. This means that a compact of Ti and Ni powders will increase some 940°C if fully reacted in an adiabatic environment. The process is therefore highly sensitive to heat transfer to the outside environment; hence, process parameters need to be closely monitored, controlled and optimized to achieve consistent material properties. As shown in Figure 1, porous Nitinol exhibits an irregular and interconnective pore structure closely resembling human bone.

The objective of this study was to examine the properties of porous Nitinol for biomedical applications. The material analysis was further performed using X-ray diffraction to elucidate the phases present. Both local and systemic corrosion tests were performed to assess the material resistance to corrosion by cyclic potentiodynamic polarization and Ni ion release test. A wicking test was performed to assess its potentially hydrophilic property by absorbing aqueous solution without exerting external forces. Preclinical sheep studies were performed to characterize biocompatibility and speed of bone formation within the porous matrix. The current study delineates the material properties of porous Nitinol and establishes preclinical evidence of demonstrating osteoconductivity, safety and biocompatibility.

## 2. Material/Methods

The combustion synthesis of porous Nitinol used an equiatomic mix of commercially available Ni and Ti powders. The titanium powder complies to the ASTM F67. The powders were blended in a mill to achieve a homogenous mixture and cold compacted in a mold to add a green strength sufficient to maintain a desired shape. The mold was placed in an Argon-purged furnace to initiate the combustion synthesis. The green compact was ignited by an electrical arc.

Compression testing on porous Nitinol was performed according to ASTM-E9. Cylindrical specimens (Ø6 mm x 12 mm) were prepared by electron discharge machining (EDM). An Instron™ machine was used to perform the test at a rate of 0.5 mm/s. Reported values represent the average of five individual tests.

Porosity and pore sizes were measured at three locations along the length of the synthesized porous Nitinol stock part. Similar to the compression specimens, cylindrical specimens with a dimension of Ø6 mm x 12 mm were machined. The surfaces were prepared with a SiC polish. The porosity was calculated using the Archimedes principle. The pore sizes were measured using a Nikon optical microscope model SMZ800 and NIS Elements D software to determine the average pore size and its standard deviation.

Differential Scanning Calorimeter (DSC) analysis was performed to determine the transition temperatures using a TA Instrument model DSCQ100 per ASTM D3418.

X-ray diffraction was performed at Evans Analytical Group (Sunnyvale, CA) to determine the phases present in the porous Nitinol specimens with an emphasis on the presence of Ni metal and Ni rich phases. Data was collected on a Bruker GADDs microdiffractometer equipped with a copper X-ray tube, incident-beam monochromator, 800-micron pinhole collimator, laser alignment system and 2D detector.

The corrosion behavior of six porous Nitinol specimens were measured by potentiodynamic polarization testing according to ASTM F2129. The rest potential (Er) and breakdown potential (Eb) were analyzed to assess the corrosion resistance. Superior resistance is characterized by higher Eb value indicating a resistance to local or pitting corrosion. In many cases the oxygen evolution potential (Eox ev) was reached prior to breakdown. The test was performed in a phosphate buffered saline solution at the temperature of 37 ± 1°C. The pH was maintained at 7.4 ± 0.1 and the scan rate was 0.167mV/s.

Ni ion release was performed on five specimens to determine the mean and standard deviation. A solution volume to implant surface area ratio of 1ml/cm^2^ was used, as specified in ISO 10993-15. An OmniPur 10x PBS Concentrate was diluted to create the PBS solution. The test was performed for approximately 60 days under static condition. The temperature of the incubator was maintained at 37 ± 1°C. The solution from each vial was analyzed using inductively coupled plasma mass spectrometry (ICP-MS) to quantify the amount of nickel released from the specimens.

A wicking test was performed on porous Nitinol, Physical Vapor Deposition (PVD) porous tantalum, and sintered porous titanium. Each specimen was EDM machined into a dimension of Ø10 mm x 30 mm. They were ultrasonically cleaned and dried in a vacuum oven. The reported values represent the average of three tests. The percentage of open porosity for each specimen was determined. The relative percentages of open and closed porosity were determined applying the Archimedes principle. The total porosity of each specimen was calculated based on the theoretical density for Nitinol, tantalum, and Ti-6V-4Al was 6.45, 16.7 and 4.42 g/cm^3^, respectively.

The wicking test was performed by filling a glass beaker with 15 ml of phosphate buffer solution. Each specimen was suspended with 2 mm of the distal end submerged in the reservoir. The difference between the volume of the reservoir before and after the test was used to calculate the solution wicked by each specimen. Measurements were taken every ten seconds up to one minute, and every minute thereafter. The test was terminated at ten minutes.

An ovine study was performed at the Surgical and Orthopaedic Research Laboratories at the University of New South Wales (UNSW) according to ISO10993-6 to assess the speed of bone ingrowth within porous Nitinol specimens. The study was conducted under approval from the Institutional Animal Care an Ethics Committee (ACEC UNSW Australia). Five skeletally mature sheep received porous Nitinol cylindrical implants with a dimension of Ø6 mm x 25 mm. A 3 cm surgical incision was made 50 mm from the articular surface along the anteromedial aspect of the tibias. The exposed periosteum was sharply dissected to expose the underlying cortical bone. Three bi-cortical 6 mm holes were prepared in the cortical bone using increasing diameter drills up to 6mm. The implants were inserted in a line to line manner. The periosteum was reflected, and the soft tissue and skin closed in layers. Euthanasia of one sheep occurred at 2-week, 4-week, 6-week, 8-week, and 12-week time points.

Specimens were placed in 10% buffered formalin and sequentially dehydrated in increasing concentrations of ethanol for embedding in polymethylmethacrylate (PMMA). Embedded implants were sectioned along the long axis of the implants using a Leica SP 1600 Microtome. Two thin (~15-20 micron) sections were cut from each embedded implant for histological evaluation. Sections were stained with methylene blue and basic fuchsin for examination under a light microscope (Olympus, Japan) for bone organization and reaction at the implant-bone interface and within the porous structure of the implant. The cortical histology was performed according to ISO 10993-6 for local tissue reactions

A separate ovine study was undertaken with an objective to assess the biocompatibility per ISO10993-6 as part of the regulatory filing and measure the performance of a porous Nitinol spinal fusion device in a functional large animal model. This study was performed at the Preclinical Surgical Research Laboratory (PSRL) and Orthopaedic Bioengineering Research Laboratory (OBRL) at Colorado State University following the approval of Colorado State University's Institutional Animal Care and Use Committee (IACUC) under Good Laboratory Practice (GLP) defined in the FDA, 21 CFR 58. Eight skeletally mature sheep (< 3.5 years of age, 60-100 kg) underwent an instrumented lumbar intervertebral fusion at L2-L3 and L4-L5 using a retroperitoneal lateral approach. Each animal was implanted with two different types of interbody fusion cages to minimize variation among the animals: control group, Zeniva PEEK cage (Eisertech LLC., 12 mm length x 14 mm width x 6 mm height) with autologous bone graft; experimental group, porous Nitinol cage (12.5 mm length x 14.5 mm width x 7 mm height) with autologous bone graft. Devices were alternately implanted and randomly chosen, with a PEEK cage (n=8) or porous Nitinol cage (N=8) at L2-L3 or L4-L5. Operated levels were stabilized with pedicle screws and connecting rods. The animals were divided into two time points on the basis of follow-up period; 4 months and 6 months. Four sheep were euthanized at each time point.

The spinal columns were removed as a unit (L1-L6), placed in neutral formalin (10%) and prepared for ground sectioning. After L2-L3 and L4-5 were separated, the samples were cleaned with acetone and infiltrated with series of resin and cured for approximately 20 days. Once cured, the specimens were polymerized into a hardened plastic block. Histology slices were taken in the sagittal plane to display the implant at the operating level and adjacent vertebral body. Slices were cut using an Exakt diamond blade bone saw to a thickness between 300 and 400 *µ*m. All sections were ground using an Exakt micro grinder to approximately 50 *µ*m and stained using Sanderson's Rapid Bone stain and counterstained using Van Gieson bone stain.

Blood samples (n=88) were collected every week for six weeks and monthly until euthanasia to analyze the blood nickel level. Whole blood samples were collected for ICP-MS dosage in EDTA tubes. EDTA tubes were filled with a minimum of 50% by volume with a blood sample and mixed by rotating eight times after filling to prevent clotting. A minimum volume of 2.5 ml was dispensed for analysis.

## 3. Results and Discussion

### 3.1. Material and Mechanical Properties

The nature of the porosity formed by the combustion synthesis is a result of the green compact porosity. When the compaction is kept to a minimum, the synthesized compound exhibit inadequate bonding. Excess compaction can cause the reaction temperature to exceed the melting temperature of the intermetallic compound, causing the pre-existing pores to collapse and large voids to form along the direction of the reaction propagation. Hence, each processing parameter needs to be carefully optimized and controlled to obtain consistent and high porosity material. As shown in Table 1, the general porosity of the optimal porous Nitinol was found to be 64% – remarkably high compared to conventional powder processes and other combustion synthesized porous Nitinol [13–15]. The material exhibits an anisotropic three-dimensional interconnected network of pores with greater than 90% of the pores (by volume) open to the surface. The anisotropy was observed from in the pattern of the pore structure where the striation was prominent perpendicular to the length of the ingot or the direction of the combustion reaction. The process condition that influences the anisotropy is under further investigation.

In this particular study, the pore size ranged from 100 to 400 *µ*m, comparable to a cancellous bone pore size of 10-500 *µ*m [19]. The osseointegration of porous Nitinol has been described in an animal model by Ayers et al. [20] where three types of porous Nitinol were implanted in cranial bone. The average pore sizes ranged between 179-353 *µ*m and the porosity between 42.9-54.4%. An average bone ingrowth of 31.1-37.9% was achieved after six weeks. Bone ingrowth was observed when the porous Nitinol specimens were press-fitted into the bone, regardless of the pore sizes. A similar amount of bone ingrowth was observed within the pore size range of 150-500 *µ*m. There are several conclusions as to what the ideal pore size is for promoting bone ingrowth, with reports of 50 *µ*m to 400 *µ*m [17, 21, 22]. Regardless of the ideal, good ingrowth has been observed within the entire range of 50 to 500 *µ*m.

**Figure 2:**
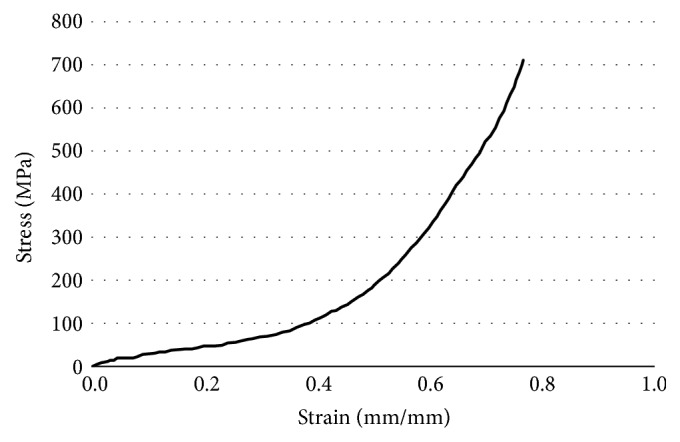
Stress-Strain curve of Ti-Ni under compression. Test was terminated due to load cell limitation.

As shown in Figure 2, a typical stress-strain curve exhibits three distinct sections: linear elasticity, a constant stress plateau, and densification [23]. The linear elastic phase exhibits a short linear region where the specimen is elastic, while the yield plateau of constant stress exhibits a gradual increase in the stress with respective to a large increase in the strain. The densification region is characterized by the coagulation of the porous structure and pore collapsing.

**Figure 3:**
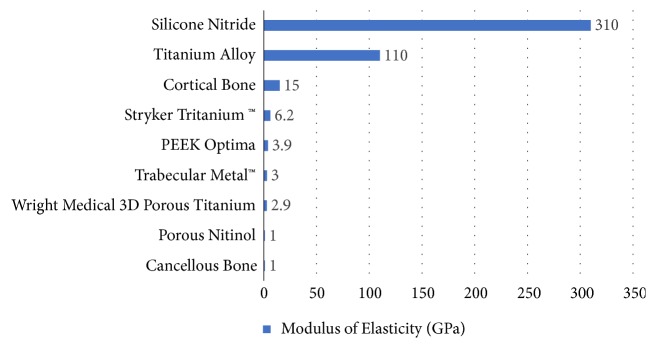
A comparison of elasticity moduli for commercially available biomaterials and cortical bone.

As shown in Figure 3, the elastic modulus of ceramic (silicon nitride) and wrought metals (titanium alloys) used in orthopedic implants is 320 and 110 GPa, respectively. Conversely, commercially available porous metals such as porous tantalum (Zimmer, Trabecular Metal™), sintered porous titanium (Wright Medical, Biofoam™), and 3D printed porous titanium (Stryker, Tritanium™), still exhibit modulus of elasticity of 3.0, 2.9, and 6.2 GPa respectively, which are at least 3 times stiffer than cancellous bone. Porous Nitinol exhibits an elastic modulus of 1 GPa, which is within the range of the stiffness of cancellous bone (~1.15 GPa) [24], potentially minimizing the effect of bone atrophy caused by stress shielding.

### 3.2. Scanning Electron Microscopy (SEM)

**Figure 4:**
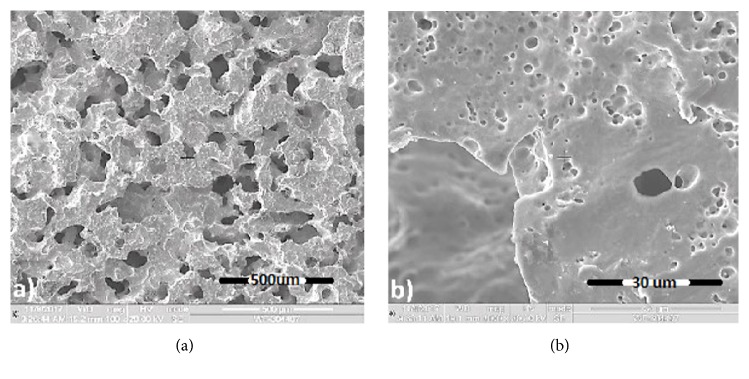
SEM survey of combustion synthesis porous Nitinol at (a) 100X and (b) 1000X magnification.

Porous Nitinol exhibits an open-celled and irregular structure critical to early osteoblast formation and vascularization. As shown in Figure 4(a), the material exhibited an anisotropic and interconnective pore structure with random variation in the pore sizes. A video of the micro CT survey can be found by going to https://youtu.be/OnQboEVYUSA. SEM survey revealed the presence of three districted pore sizes: macro-, micro-, and nanoscale, which may serve as a platform for osteoblast anchorage and cell proliferation (Figure 4(b)). Nanoscale porosity might facilitate early cell migration and adhesion while micro- and macroscale porosity promotes osteoblast proliferation and fixation on various pore structures similar to that found naturally in cancellous bone [25–27]. Conversely, 3D printed porous metals fabricated by additive manufacturing exhibit smooth surfaces without nanoscale porosity. Furthermore, porous Nitinol exhibits a rough nanotextured surface topography not found in the conventional porous metals produced by additive manufacturing and do not exhibit loosely fused or unbounded particles which may produce debris and obstruct the growth of osteoblasts [27].

### 3.3. Transformation Temperatures

**Figure 5:**
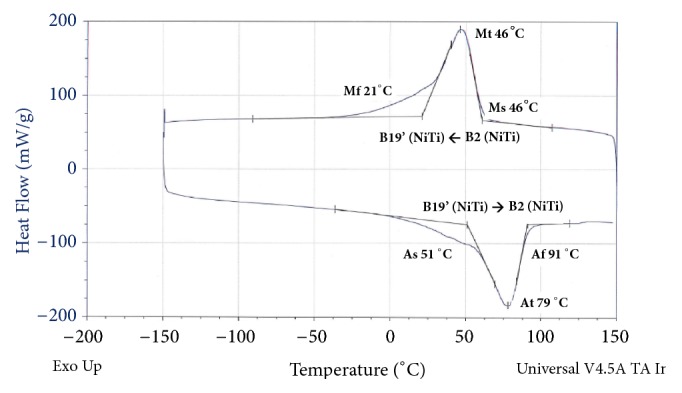
DSC of porous Nitinol indicating an equiatomic or titanium-rich matrix phase.

The phase transition temperatures were determined by DSC. As shown in Figure 5, the material exhibits no R-phase, but rather a single-step forward and backward transformation between the high temperature austenitic phase (B2) and the martensitic phase (B19') with the M_f_ and A_f_ temperature of 21°C and 91°C, respectively. This is consistent with transformation temperatures one would expect from an equiatomic composition. Successively, this is a beneficial property as it could demonstrate compliance with the interfacial surfaces.

### 3.4. X-Ray Diffraction (XRD)

**Figure 6:**
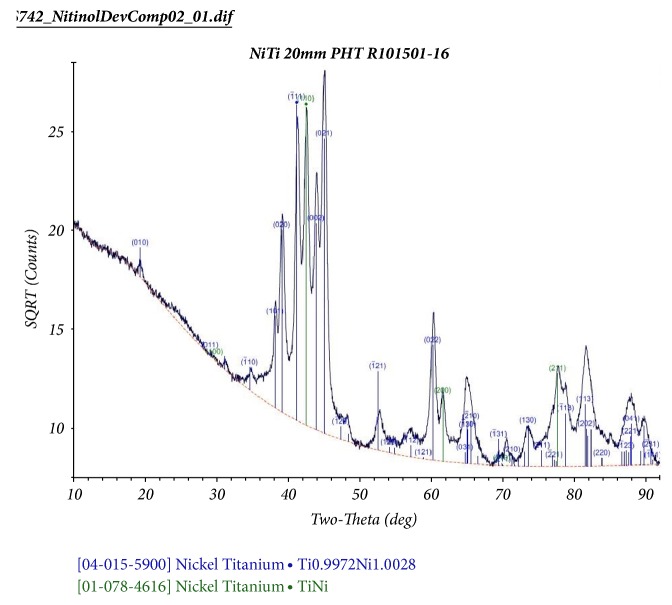
The XRD spectrum is consistent with a mixture of Martensite and Austenite, and lacks signature peaks from other intermetallic compositions.

Three specimens were analyzed using XRD to determine the crystal lattice structures with an emphasis on determining the presence of Ni-rich phases, as shown in Figure 6. Two stable intermetallic Ni-rich compounds are found between pure nickel and the NiTi itself: Ni_2_Ti and Ni_3_Ti_2_. A third metastable phase (Ni_4_Ti_3_) can also be found in aged conditions. The concern with residual Ni-rich phases would be a potentially greater tendency to release nickel. The XRD scans, however, exhibit only the monoclinic (Spatial Group: P21/m (11)) and cubic forms of TiNi (Spatial Group: Pm-3m (221)) phases, with little or no difference between the three samples. The lack of evidence of any Ni-rich phases indicates that diffusion rates were sufficient to complete the NiTi formation reaction.

### 3.5. Corrosion

**Figure 7:**
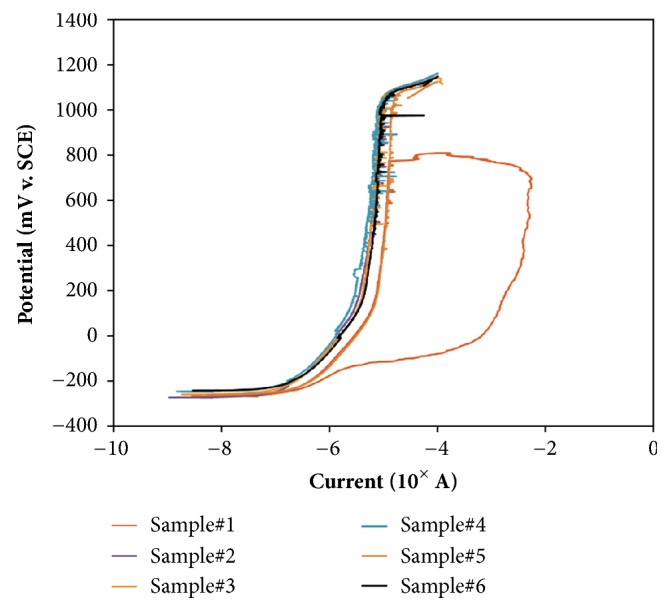
Potential vs. current curves for combustion synthesis porous Nitinol.

Six specimens were tested to assess their resistance to pitting corrosion by potentiodynamic polarization testing per ASTM F2129. Both the forward and a backward scan are shown in Figure 7. The noise on the potentiodynamic curve is most likely caused by the porous structure and its rough surface. As shown in Table 2, five specimens reached oxygen evolution without the breakdown of the oxide layer while one specimen experienced breakdown at 772 mV with protection potential at -236 mV. The test results show the high pitting corrosion resistance of porous Nitinol that are completely comparable to wrought material.

What makes porous Nitinol unique is its high resistance to corrosion. While the ASTM F2129 does not have an acceptance criterion, literature has resorted the acceptance categories to three (3) distinct regions which are commonly used to assess the corrosion resistance. Rosenbloom and Corbett (2007) reported with breakdown potentials below 300 mV is considered unacceptable [28]. Material that exhibits breakdown potential below 600mV, but above 300 mV would be considered marginal and more testing may be needed to assess the material performance under the indicated use in the biological environment. Breakdown potential above 600mV is regarded as resistance to corrosion.

Li et al. (2003) [29] tested the corrosion resistance of porous Nitinol with a porosity of 55.1 ± 1% using the nominal surface area of 1cm^2^ and recorded a breakdown potential of 137 ± 115 mV. Porous Nitinol has historically been shown to exhibit less corrosion resistance [30], while others have reported a breakdown potential of 270 ± 70 mV with porous Nitinol material with a porosity of 65 ± 10% [31]. Based on the criteria set by Rosenbloom and Corbett (2007), the said material from the literature which exhibited a breakdown potential lower than 300 mV would be susceptible to pitting corrosion under a physiological environment. The porous Nitinol tested in the current study exhibited a porosity of 64 ± 1 % with a significantly higher breakdown potential of 772 mV on 1 test specimen while five other test specimens reached oxygen evolution, discerning that pitting corrosion is unlikely to occur. While porous materials may share a similar design and/or property, the author advises for the individual to fully recognize the material performance differences even within one particular material such as the porous Nitinol.

### 3.6. Ni Release

**Figure 8:**
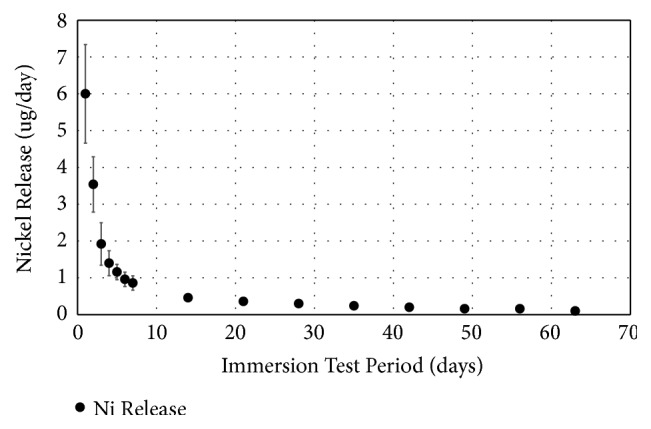
Ni ion release of the porous Nitinol for 63 days. Specimens exhibited Ni ion release below the acute tolerable limit of 70 *µ*g/day for period up to the first 24 hours and below the long-term tolerable limit of 35 *µ*g/day beyond the 1st day.

Five specimens were tested for Ni release as shown in Figure 8. On the 1st day, the average was 6.0 ± 1.2 *µ*g/day with the maximum ion release for a single specimen of 7.6 *µ*g/day. The Ni release followed a decreasing trend, reaching 1 *µ*g/day on the 6th day and to 0.2 *µ*g/day at the 35th day.

One of the potential challenges of metallic implants is its potential susceptibility to corrosion. Corrosion can be detrimental to the implant performance and may release metal ions that could be harmful to the body. The local corrosion is the effect of bulk material breaking down due to electrolysis, forming pits where fissures can propagate, resulting to implant failures. In parallel, metallic implants can still release metal ions. Excess exposure to Ni ion can lead to cell necrosis.

Cytotoxic effects were reported when exposed to materials containing nickel [32–34]. Therefore, CS porous Nitinol with resistance to Ni leaching is crucial to its biocompatibility and use as implants. As suggested from the FDA, based on the acute and chronic tolerable limits (Tl) for nickel ion release for eliciting cytotoxic effect on an adult is based on the value reported by Sundermann (1983) with the short-term (<24hrs) parenteral tolerable limit (Tl) for Ni is 10 *µ*g/kg/day and the long-term (>24hrs) parenteral tolerable limit (Tl) for Ni is 5 *µ*g/kg/day [35, 36]. The tolerable limit for the first 24 hours is 70 *µ*g/day and anything longer than the first 24 hour is 35 *µ*g/day. While there are no recognized acceptance criteria for acceptable nickel ion release, the use of both short and long-term parenteral TI ensures the cytocompatibility of the test material.

### 3.7. Wicking Properties

**Figure 9:**
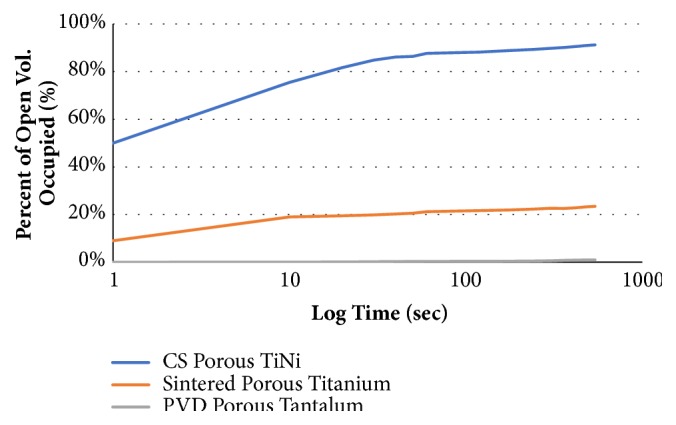
Porous Nitinol, Sintered porous Ti, and PVD porous Ta wicking PBS solution as a percentage of open pore volume over 10 minutes.

As shown in Table 3, the total amount of solution wicked by the three materials varied greatly. Porous Nitinol, sintered porous Ti and PVD porous Ta wicked 91.2%, 23.6% and 0.9% of the overall percentage of the open volume at the duration of the test, respectively. As shown in Figure 9, porous Nitinol exhibited exceptional wicking, achieving approximately 78% of the open volume wicked within 10 seconds, while sintered porous Ti and PVD porous Ta only exhibited 20%, and 0.9% absorption, respectively. It is astonishing to observe that most of the wicking occurred within the first 10 seconds of the experiment. A video of the wicking demonstration can be found by going to (https://youtu.be/tSTaYY1_2kM).

Porous Nitinol exhibited exceptional wicking properties absorbing faster and more volume than the other porous metals tested. PVD porous Ta exhibited an almost hydrophobic behavior in comparison. The exact cause for this remarkable behavior is unclear, though likely due to combination of the pore characteristics and the chemical make-up of the porous Nitinol material. Regardless of the mechanism of wicking, it appears likely that it is this wicking behavior that results in the extraordinary bone ingrowth response of porous Nitinol. The material's random pore variations and the highly interconnective open pores may be beneficial for transferring nutrients and fluids which aids in early vascularization, cell response and osseointegration which are crucial for biomedical applications.

### 3.8. Biocompatibility

**Figure 10:**
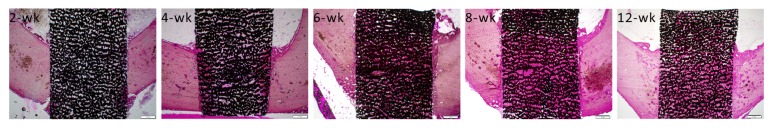
Histology at 2, 4, 6, 8, and 12 weeks demonstrates the continual progression of new bone integration and remodeling within the porous domains of the implant.

Using five sheep, cylindrical implants (n=30) with a dimension of Ø6 mm x 25 mm were placed in three bi-cortical sites along the anteromedial aspect of the tibias. The implants were inserted in a line to line manner. Euthanasia of one sheep was performed at each time point; 2, 4, 6, 8 and 12 weeks. Rapid osseointegration into the porous domains of the porous structure was noted as early as 2 weeks, and continual progression of new bone formation into the porous domains of the implant improved with time. Complete bone ingrowth across the implant from one cortex to the other in the medial and lateral sites was noted as early as 6 weeks. All implants at 8 and 12 weeks demonstrated comprehensive bone growth throughout the porous structure. At 8 and 12 weeks, interestingly, new bone formation was seen growing up the implant into the medullary canal demonstrating the extraordinary osteoconductive nature of the porous Nitinol used in this study. As seen in Figure 10, bone appeared to walk through and up the porous implants in a continuous manner.

No adverse reactions were noted in any site in any animal. The underlying response to the surgical procedure was normal. There were no adverse reactions to the organs. The harvested bones were further evaluated using macro-zoom photography. The cortical sites were carefully inspected for any evidence of adverse reactions. No evidence of any adverse reaction was noted at any time point in any animal.

**Figure 11:**
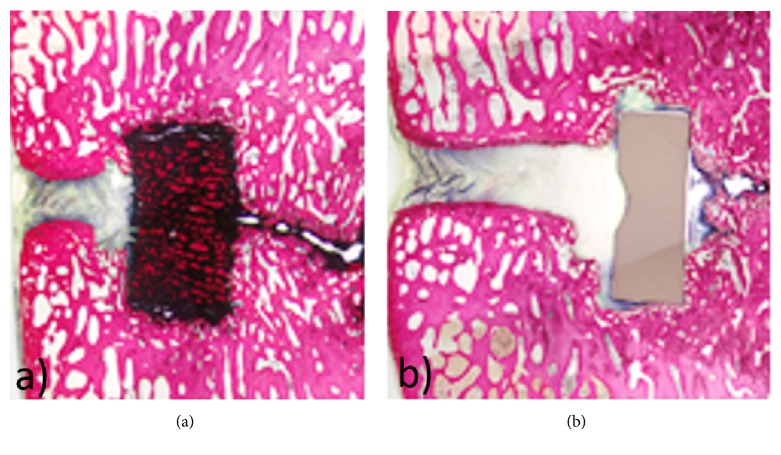
4-month histological analysis of (a) porous Nitinol and (b) PEEK device. Bone growth throughout the pores of porous Nitinol and fibrous tissue encapsulation around solid PEEK.

Using a lumbar interbody fusion model in eight sheep, histological slices were taken in the sagittal plane to display the implant at the operated level and adjacent vertebral bodies. All samples demonstrated new reactive bone completely capping the vertebral endplates adjacent to the implant at 4-month and 6-month time points. Porous Nitinol implants had favorable bone directly in contact with the implant surface (bone apposition) whereas PEEK exhibited more fibrous tissue encapsulation around the implants surface, thus, preventing bone attachment in many cases. At both 4 and 6 months, porous Nitinol had a single implant with bone apposition values of 72.58% and 77.48%, respectively. At 4 months, two of the porous Nitinol implants had 100% of their interconnected pores filled with reactive new bone, fibrous reactive tissue, and osteoid. Proportion of the filling by these tissue types was variable between individuals and varied within portions material. As seen in Figure 11, pores throughout the structure are completely filled with new bone at 4 months.

**Figure 12:**
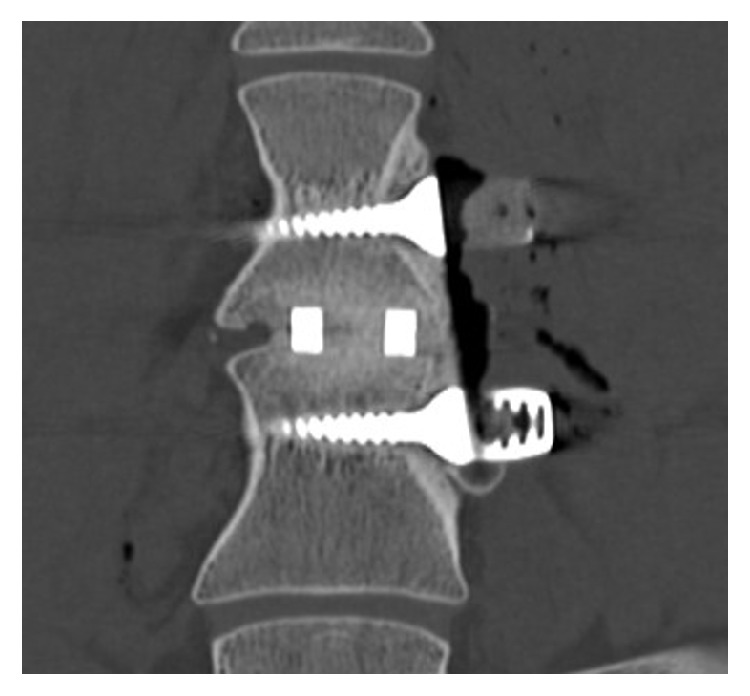
4-month CT scan survey of explanted combustion synthesis porous Nitinol material exhibiting complete through growth of bone after four months of implantation in an ovine study.

Computed tomography (CT) examination confirmed no implant migration occurred throughout the study and allowed visualization of fusion through the central cavity along with intimate bone-to-implant contact, as shown in Figure 12. The extraordinary properties of porous Nitinol may promote early vascularization and bone growth through the porous matrix inducing firm fixation between bone and implants.

Histological analysis of soft tissues (N=42) was performed per ISO 10993-6 on heart, liver, lung, spleen, kidney and lymph node were normal for conventionally raised sheep. There were no changes that were considered as potential toxic effects of the implants or procedures.

**Figure 13:**
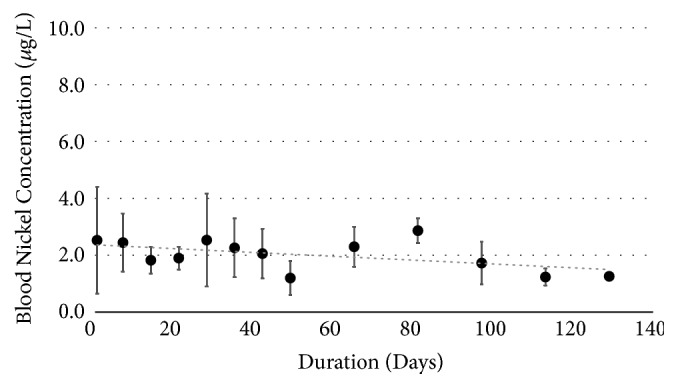
Ovine nickel blood level over six months exhibiting a decreasing trend.

Nickel analysis on blood samples (n=88) was performed to confirm the low nickel-release rates measured* in-vivo*.  Figure 13 shows a slight decreasing trend based on the linear regression. Preoperative nickel blood levels in sheep were determined to be 2.5 ± 1.9 *µ*g/L. The postoperative nickel blood level prior to euthanasia was 1.3 ± 0.1 *µ*g/L. There was neither a significant increase nor decrease in nickel blood levels over the course of 6 months. The nickel blood level also seems to exhibit a cyclic pattern over the course of the analysis.

The two ovine studies demonstrate osseointegration and biocompatibility which delineate the potential advantages of early bone ingrowth and safety when using porous Nitinol as an orthopedic implant. The authors believe that the exceptional bone ingrowth of the porous Nitinol analyzed may be attributed from the combination of unique properties that encompass (1) load sharing from matching the stiffness of the surrounding cancellous bone, (2) irregular porous structure eliciting various sizes from macropores to nanopores, (3) optimal pore size and porosity, (4) nanotextured surface roughness, (5) titanium oxide surface chemistry, and (6) the exceptional wicking performance.

## 4. Conclusion

Porous Nitinol produced by combustion synthesis exhibits a unique combination of material properties which hold promise for orthopedic implants that interface a bony environment. The inherent rough external surfaces elicit acute fixation and mitigate the risk of migration. The material has a high compressive strength and an exceptionally low compressive modulus closely matching that of cancellous bone. Perhaps most startling is the remarkable wicking behavior of the porous Nitinol material, presumably due to the interconnected open pore structure and surface chemistry. Moreover, the pores size and distribution closely match that of human bone, which is critical for cell attachment, early vascularization, and rapid bone formation. The diffusivity of nickel and titanium were found to be sufficient to avoid measurable formation of Ni-rich compounds, resulting in exceptional corrosion and Nickel release characteristics. The current study delineates the material properties of CS porous Nitinol and establishes preclinical evidence demonstrating an osteoconductuve bone substitute superior to alternative commercially available porous tantalum and porous titanium materials.

Based on the above, the FDA has recently approved the first porous Nitinol implant produced via combustion synthesis, and human implantation has begun with exceptional physician feedback on implant perfomance and speed of patient recovery.

Attention now needs to be turned to examining whether the process can be controlled tightly enough to produce superelastic properties and to examine applications exposing the implants to loading modes other than compression.

## Figures and Tables

**Table 1:** Average pore size and porosity of CS Ti-Ni within the range of cancellous bone.

	CS Porous Ti-Ni	Cancellous Bone	Pore Size Range for Bone Growth
Pore Size(*µ*m)	235	200-300 [16]	50-500 [17, 18]
Std. Dev.	143	-	-
Porosity (%)	64	45.3-69.8	-
Std. Dev.	1	-	-

**Table 2:** Corrosion behavior of combustion synthesis porous Nitinol.

**Sample #**	**Er** **(mV v. SCE)**	**Eb** **(mV v. SCE)**	**Eox, ev** **(mV v. SCE)**	**Ep** **(mV v. SCE)**
1	-266	772	N/A	-236
2	-270	N/A	1060	N/A
3	-257	N/A	1060	N/A
4	-244	N/A	1050	N/A
5	-257	N/A	1060	N/A
6	-239	N/A	1030	N/A

**Average**	-256	772	1052	-236
**Std. Dev.**	12	N/A	13	N/A

**Table 3:** Wicking characteristics of porous material after a 10-minute immersion.

**Specimen**	**Porosity**	**Open Porosity**	**Percent Volume Wicked **
porous Nitinol	66.7%	90.0%	91.2%
Sintered Porous Ti	61.8%	88.2%	23.6%
PVD Porous Ta	67.2%	80.4%	0.9%

## Data Availability

The data on porous Nitinol used to support the finding of this study are available from the corresponding author upon request.
